# The value of ultrasound in transradial access cerebral angiography

**DOI:** 10.3389/fneur.2025.1715218

**Published:** 2025-12-01

**Authors:** Zhen Sun, Wenyue Wei, Congcong Jia, Hui Zhang, Chen Zhou, Gang Luo, Wanying Li, Simin Li, Yuhong Wang, Shuming Guo

**Affiliations:** 1 Department of Neurology, Linfen Central Hospital, Linfen, Shanxi, China; 2 Clinical College of Neurology, Neurosurgery and Neurorehabilitation, Tianjin Medical University, Tianjin, China; 3 Department of Neurology, Tianjin Key Laboratory of Cerebrovascular and Neurodegenerative Diseases, Tianjin Dementia Institute, Huanhu Hospital Affiliated to Tianjin Medical University, Tianjin, China; 4 Beijing Institute of Brain Disorders, Capital Medical University, Beijing, China; 5 Department of Neurological Intervention Center, Beijing Tiantan Hospital, Capital Medical University, Beijing, China; 6 Shanxi Medical Service Evaluation Center, Department of Health Management, Taiyuan, Shanxi, China

**Keywords:** ultrasound, transradial access, cerebral angiography, radial artery variations, complication

## Abstract

**Background:**

Transradial access (TRA) for cerebral angiography presents challenges such as puncture failures, prolonged procedure times, and difficulties in catheter advancement due to radial artery variations. Utilizing ultrasound guidance, which has demonstrated success in coronary angiography, may improve TRA outcomes. This study aimed to assess the efficacy of ultrasound-guided TRA in cerebral angiography, focusing on success rates, procedure duration, and patient satisfaction.

**Methods:**

A prospective, non-randomized, controlled trial included 197 patients scheduled for TRA between June 2022 and January 2024. Patients undergoing cerebral angiography through TRA were divided into control and ultrasound group. The ultrasound group underwent preoperative right upper extremity artery ultrasound assessment, with ultrasound-guided puncture for challenging cases. The primary outcomes were procedure completion rate, duration, patient satisfaction, and complication rates. Statistical analyses were performed using Student’s t-test for continuous variables and the chi-square test for categorical variables to compare these outcomes between the two groups, using a two-tailed significance level of *p* < 0.05.

**Results:**

This study included a total of 197 patients: 73 in the control group and 124 in the ultrasound group. TRA was completed in 69 patients (94.5%) in the control group and 117 patients (99.2%) in the ultrasound group. The average operation time was 0.67 ± 0.19 h in the control group and 0.55 ± 0.19 h in the ultrasound group (*p* < 0.001). Difficult access occurred in 15 patients (21.7%) in the control group compared with 2 patients (1.7%) in the ultrasound group (*p* < 0.001). No significant differences were observed in either complication rates or patient satisfaction: 8 patients (11.6%) in the control group and 9 (7.7%) in the ultrasound group (*p* = 0.372) had complications, while 57 patients (82.6%) in the control group and 109 (93.2%) in the ultrasound group reported satisfaction (*p* = 0.089).

**Conclusion:**

Ultrasound-guided TRA in cerebral angiography effectively mitigated challenges related to puncture failures and procedure duration, offering the potential to improve outcomes and patient satisfaction in TRA procedures.

## 1 Introduction

Transfemoral arterial access (TFA) remains a conventional approach for cerebral angiography, favored for its straightforward puncture technique and ease of distal vessel selection. However, TFA is associated with a higher incidence of puncture site complications, primarily due to the deep anatomical location of the femoral artery (1). This depth complicates effective post-procedural compression, increasing hemostasis difficulty and inguinal hematoma risk (reported in about 4.2% of TFA cases) (2). Additionally, prolonged femoral artery compression postoperatively may cause back pain, urinary retention, and deep vein thrombosis, impairing patient satisfaction and recovery. While ultrasound-guided TFA can reduce puncture failure, it has limitations, particularly in patients with obesity or arterial calcification, where pseudoaneurysm and other complications may persist (3). In contrast, the radial artery’s superficial location makes TRA a safer alternative, demonstrating a 60% reduction in access site complications compared to TFA. This approach facilitates easier compression, minimizes major bleeding risk, and reduces the need for prolonged immobilization, leading to shorter hospital stays and lower costs (4–6). Moreover, TRA is associated with shorter fluoroscopy times, higher patient satisfaction, and fewer complications (7–11). For neuro-interventionalists, proficiency in TRA is typically achieved after a learning curve of 30–50 cases, making it feasible for widespread adoption (3, 8, 12, 13). Beyond this threshold, procedure times and success rates consistently rival or exceed those of TFA. Over the past decade, these advantages have made neuro-interventionalists increasingly interested in TRA for both diagnostic and therapeutic procedures (14–18).

Although TRA offers several benefits, it has also faced challenges, including a notable 13.8% incidence of abnormal radial artery dissection. This issue significantly heightens the risk of procedural failure. Additionally, the study found that the risk of surgical failure in cases with radial artery variations is markedly higher, at 14.2%, compared to just 0.9% in those with normal radial arteries (19, 20). If severe tortuosity of the subclavian artery or arterial spasm clasping the contrast catheter is encountered it can greatly increase the difficulty of the procedure (21, 22). The incidence of radial-femoral conversion was reported by Tso et al. (7) to be 1.2% (7/607), which is higher than the incidence of femoral-radial conversion at 0.3% (2/635). Patients requiring conversion from TRA to TFA undergo additional discomfort and increased procedure time and costs. How to improve the success rate of TRA surgery and reduce the risk of surgery are urgent problems to be solved.

Notably, Bernat et al. discovered that using ultrasound guidance for radial artery cannulation in coronary angiography greatly reduced puncture failure decreased the number of attempts, and improved efficiency. The approach also minimizes the risk of radial artery spasms and alleviates pain and swelling (23). This study aimed to determine whether ultrasound assistance could address similar challenges encountered in TRA during cerebral angiography, specifically by improving success rates, shortening procedure times, and enhancing patient satisfaction through preoperative ultrasound evaluation and guidance during difficult punctures. Currently, TRA utilizes the Simmons series of contrast catheters, which are designed based on TFA. This study aimed to gather data on TRA access routes to aid in the development of TRA-specific catheters.

## 2 Methods

### 2.1 Study design and population

This prospective, non-randomized, controlled trial was conducted in the neurology unit of a 2,100-bed hospital. It involved sequential data collection from patients undergoing diagnostic cerebral angiography via TRA from June 2022 to January 2024. All patients were divided into two cohorts based on their personal preference for pre-procedural assessment: a control group and an ultrasound group. Inclusion criteria were as follows: (1) individuals aged 18–90 years; (2) patients needed diagnostic TRA for cerebral angiography. Exclusion criteria: (1) comorbid major psychiatric or systemic illnesses; (2) a negative Allen test result; (3) a blood pressure discrepancy of more than 20 mmHg between the upper extremities; (4) with a history of iodine allergy. Informed consent form had been obtained from all participants, and the study protocol was approved by the Ethics Committee of Linfen Central Hospital (Ethics 2022-23-1).

### 2.2 Clinical data collection

Comprehensive demographic and clinical data were collected from the medical record system, including age, sex, height, weight, and medical history. Laboratory parameters, such as hematologic profiles, glucose and lipid levels, and liver and renal function tests, were documented. The time from arterial puncture initiation to sheath removal, postoperative satisfaction, surgical complications, and measurements of the aortic arch and its branch vessels were meticulously recorded.

### 2.3 Preoperative assessments

Before the procedure, bilateral upper arm blood pressure was measured, and the collateral circulation of the hand was assessed with the Allen test. In the ultrasound group, a specialized ultrasound physician examined the right upper-limb artery using a Philips EPIQ 7C color ultrasound device (Philips Healthcare, Washington, United States) equipped with a L12-3 line array probe, operating at 32–40 Hz with a mechanical index of 1.3.

### 2.4 Cerebral angiography procedures

The patient was positioned flat with the right arm rotated backward and resting on an arm board. The right radial region was sterilized and prepared in the standard manner. Puncture was performed 1 cm proximal to the distal palmar wrist crease using a subcutaneous injection of lidocaine and a modified Seldinger technique with either a 5°F or 6°F radial artery sheath (Merit Medical). Contrast was administered to verify the positioning and visualize the radial artery. A cocktail of 200 μg nitroglycerin and 3,000 units of heparin was administered to prevent spasm and occlusion. A 100 cm Simmons 2 catheter (Terumo) was advanced along a 150 cm 0.035″ J-type guidewire (INT Medical) with continuous 0.9% sodium chloride flushing. A pigtail catheter combined with a long guidewire exchange technique was used to assist in shaping Simmons. Selective arteriography of the aortic arch, bilateral common carotid arteries, and bilateral subclavian arteries was performed in each patient. If additional elective access to the internal carotid or vertebral arteries or 3D angiography was required, the corresponding operating time was subtracted to facilitate a comparison between the two groups. After angiography, a tourniquet was applied to the puncture site for patency and hemostasis and removed gradually over 6 h. Radial artery patency was assessed by palpation. Two experienced neurointerventionalists supervised all procedures.

### 2.5 Ultrasound-guided puncture

Ultrasound-guided puncture may be considered in the ultrasound group in cases of difficult access (≥ 5 punctures or puncture time ≥5 min). If radial arterial puncture fails or is unable to access, two groups explore alternative vascular puncture sites (e.g., right femoral artery). When encountering difficulties with the radial artery loop guidewire passage, it was substituted with a 0.035-inch ultra-smooth guidewire (Radifocus, Terumo) or a 0.014-inch guidewire (Anyreach C, APTMedical) to bypass the loop and straighten the radial artery. In cases of vasospasm, intra-arterial verapamil or nitroglycerin therapy was added to the initial dose.

### 2.6 Statistical analysis

We performed the Shapiro–Wilk test to assess normality for all continuous variables. Normally distributed variables were presented as mean ± standard deviation (SD). We employed the Levene test for equality of variances and the Student’s *t*-test to compare means between groups when assumptions of normality and homogeneity of variances were met. Categorical variables were reported as percentages and analyzed with the chi-square test. All analyses were conducted using SPSS 26.0 (IBM Corp., Armonk, United States), applying two-tailed tests with a significance level set at *p* < 0.05.

## 3 Results

### 3.1 Role and findings of preoperative ultrasound

**Figure 1:**
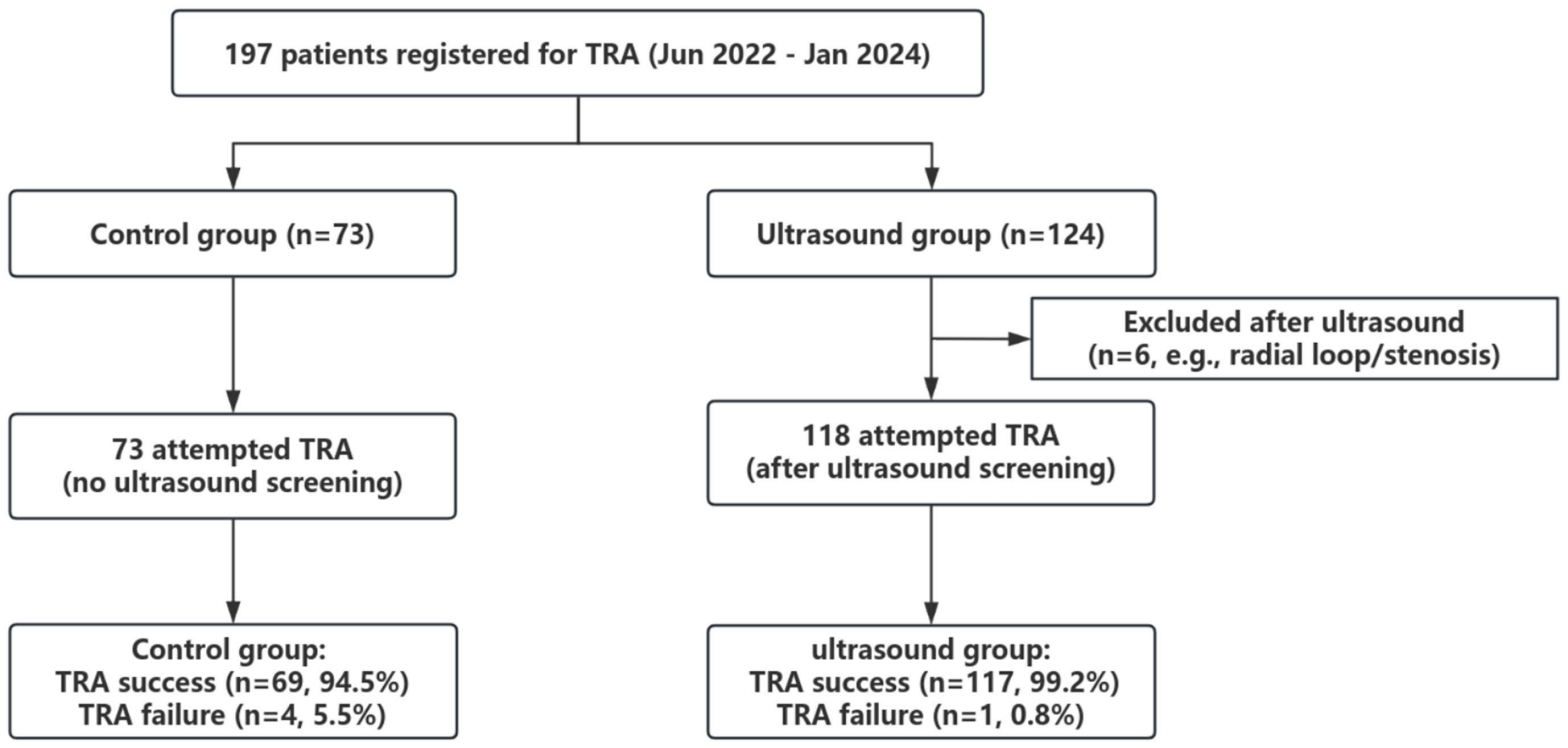
Study flowchart of sample selection. TRA, transradial access.

Seventy-three patients opted for the control group, while 124 patients chose the ultrasound group. The ultrasound group underwent ultrasonographic examination of the radial, brachial, axillary, and subclavian arteries before the procedure, and ultrasound guidance was used for puncture in cases of difficult arterial access. It is reported that the mean radial artery width was 2.3 ± 0.4 mm; however, findings that necessitated switching to the TFA included a right radial artery diameter ≤1.6 mm, the presence of a radial loop or thrombus, and a high origin of the radial artery with a diameter ≤1.8 mm. Based on these criteria, ultrasound screening was conducted on 124 patients. The results showed three cases of intact radial artery rings, one case of a high origin of the radial artery with stenosis, one case of severe radial artery stenosis, and one case of radial artery thrombosis (with coronary angiography performed a week earlier). These six patients, who faced more challenging and high-risk conditions for TRA, were preemptively transitioned to TFA. Consequently, 73 patients in the control group and 118 patients in the ultrasound group ultimately underwent TRA (refer to Figure 1).

### 3.2 Baseline characteristics

Ultimately, TRA was successfully completed in 69 of 73 patients (94.5%) in the control group and 117 of 118 patients (99.2%) in the ultrasound group. The clinical characteristics of the 186 patients who successfully underwent TRA are shown in Table 1. In the control group, there were 69 patients, with 76.8% being men. The ultrasound group consisted of 117 patients, with 75.2% being men. No significant variances were observed in demographics, medical history, or blood test results between the control and ultrasound groups.

**Table 1:** Baseline characteristics of patients undergoing TRA.

Characteristics	Total (*n* = 186)	Control group (*n* = 69)	Ultrasound group (*n* = 117)	*p*
Demographics				
Male, %	141 (75.8%)	53 (76.8%)	88 (75.2%)	0.806
Age, year	58.86 ± 11.14	60.74 ± 11.82	57.75 ± 10.66	0.078
Occupation is manual labor, %	102 (54.8%)	33 (47.8%)	69 (59.0%)	0.140
Body mass index, kg/m2	25.82 ± 3.47	25.45 ± 3.36	26.05 ± 3.54	0.258
Medical history				
Hypertension, %	131 (70.4%)	54 (78.3%)	77 (65.8%)	0.072
Diabetes mellitus, %	52 (28.0%)	16 (23.2%)	36 (30.8%)	0.266
Cerebral infarction, %	136 (73.1%)	51 (73.9%)	85 (72.6%)	0.851
Drinking, %	53 (28.5%)	17 (24.6%)	36 (30.8%)	0.371
Smoking, %	85 (45.7%)	30 (43.5%)	55 (47.0%)	0.641
Blood test results				
White blood cells, 10^9^/L	7.01 ± 2.62	6.78 ± 2.26	7.15 ± 2.82	0.351
Red blood cells, 10^12^/L	4.88 ± 3.39	4.61 ± 0.44	5.04 ± 4.28	0.411
Hemoglobin, g/L	143.89 ± 14.76	143.87 ± 13.91	143.91 ± 15.35	0.987
Platelets, 10^9^/L	233.91 ± 71.35	225.55 ± 66.83	238.84 ± 74.02	0.222
Blood urea nitrogen, mmol/L	5.56 ± 1.66	5.28 ± 1.19	5.73 ± 1.87	0.078
Serum creatinine, μmol/L	70.07 ± 15.57	68.23 ± 13.11	71.16 ± 16.88	0.218
Blood glucose, mmol/L	6.12 ± 1.88	5.88 ± 1.68	6.26 ± 1.99	0.189
Total cholesterol, mmol/L	4.66 ± 3.63	4.41 ± 1.10	4.80 ± 4.51	0.486
Triglyceride, mmol/L	1.72 ± 1.20	1.69 ± 1.16	1.74 ± 1.23	0.775

### 3.3 Comparison of operation time and operation difficulty

Corresponding to the success rates, the control group had 4 TRA failures (5.5%) among 73 patients, due to failed puncture (*n* = 2), complete radial loop (*n* = 1), spasm and stabbing (*n* = 1). Among these 4 cases, one female patient experienced severe spasm and stabbing that led her to request termination, including TFA. The others were transferred to TFA. In the ultrasound group, only 1 of 118 patients (0.8%) had a TRA failure, which was caused by a complete radical loop that was not detected during the preoperative ultrasound assessment. As shown in Table 2, the operation duration in the ultrasound group was shorter than that in the control group (mean: 0.55 h vs. 0.67 h, *p* < 0.001). The incidence of difficult access (defined as ≥5 punctures or puncture time ≥5 min) was significantly lower in the ultrasound group than in the control group [1.7% (2/117) vs. 21.7% (15/69), *p* < 0.001]. TRA-related complications included large subcutaneous petechiae, postoperative pain and discomfort at the puncture site, bleeding from ruptured vessels, and weak postoperative radial artery pulses. Overall, the complication rates were not significantly different for the two procedures [11.6% (8/69) for control group vs. 7.7% (9/117) for ultrasound group, *p* = 0.372]. Patient satisfaction levels in the ultrasound group exceeded those in the control group [93.2% (109/117) vs. 82.6% (57/69)], though the difference was not statistically significant (*p* = 0.089).

**Table 2:** Comparison of operation time and operation difficulty.

Variables	Total (*n* = 186)	Control group (*n* = 69)	Ultrasound group (*n* = 117)	*p*
Completion of TRA cases				
Operation time, h	0.59 ± 0.20	0.67 ± 0.19	0.55 ± 0.19	<0.001
Difficulty of access, %	17 (9.1%)	15 (21.7%)	2 (1.7%)	<0.001
Complication of TRA, %	17 (9.1%)	8 (11.6%)	9 (7.7%)	0.372
Degree of satisfaction, %				0.089
Satisfied, %	166 (89.2%)	57 (82.6%)	109 (93.2%)	
Mostly satisfied, %	15 (8.1%)	9 (13.0%)	6 (5.1%)	
Dissatisfied, %	5 (2.7%)	3 (4.3%)	2 (1.7%)	

### 3.4 Measurement data that may influence the TRA process

Among the 191 patients who underwent TRA, the distribution of aortic arch types was as follows: 94 type I (49.2%), 50 type II (26.2%), 37 type III (19.4%), 8 bovine (4.2%), and 2 other types (1.1%). The angle of inflection of the subclavian artery is 100.2 ± 25.3°. The angle of inflection of the subclavian artery — the angle between the portion of the subclavian artery that connects to the axillary artery and the portion that connects to the cephalic trunk artery, which is usually one of the portions of the catheter that requires the greatest amount of catheter bending during TRA angiograms and which is the site where catheter kinking most often occurs. The length of the cephalic trunk arteries is 2.4 ± 1.0 cm; the distance from the brachiocephalic artery to left subclavian artery is 3.0 ± 0.7 cm; and the distance from the subclavian inflection point to aortic arch is 4.6 ± 1.1 cm (Table 3).

**Table 3:** Characteristics of the aortic arch and its branch vessels.

Characteristics	Total
Completion of TRA cases	*n* = 191 (%)
Type of aortic arch	
I	94 (49.2%)
II	50 (26.2%)
III	37 (19.4%)
Bovine aortic arch	8 (4.2%)
Others	2 (1.1%)
The angle of inflection of the subclavian artery, °	100.2 ± 25.3
Length of cephalic trunk arteries, cm	2.4 ± 1.0
Distance from brachiocephalic artery to left subclavian artery, cm	3.0 ± 0.7
Distance from subclavian inflection point to aortic arch, cm	4.6 ± 1.1

## 4 Discussion

The results of this prospective study demonstrated that the application of ultrasound in TRA cerebral angiography significantly optimizes the surgical procedure, characterized by reduced surgery time, decreased access difficulty, and improved patient satisfaction. This improvement is particularly crucial, as it addresses the key anatomical challenges of radial artery puncture. The primary challenge is the vessel’s small diameter. As Seto et al. (24) noted, its diameter (2.4–2.6 mm) is close to the tactile discrimination threshold (2–4 mm), making vascular puncture the most demanding step of TRA. This is exacerbated in our Han Chinese cohort, with an even smaller mean diameter of 2.3 ± 0.4 mm. Beyond this fundamental constraint, anatomical variations such as high-bifurcation origins, radial artery loops, further compound anatomical challenge. Brunet et al. (13) reported that anatomical variants creating longer, narrower arterial paths are associated with higher puncture failure rates. Additionally, radial artery loops require modification for procedure completion, with risks of spasm, perforation, branch vessel avulsion, and other complications during guidewire insertion or loop curvature reduction (13). The clinical impact of these challenges is clear. A previous multivariate logistic regression analysis revealed that the number of punctures was a significant predictor of spasm (24), indicating that puncture difficulty is not merely a technical issue but a critical determinant of a major clinical complication. In this context, the role of ultrasound is twofold. First, it serves as a decisive technical solution. Our data confirm that the application of ultrasound reduced the average procedural duration by 7.2 min, indicating improved efficiency. Furthermore, ultrasound decreased the proportion of cases requiring five or more punctures or with a puncture time exceeding 5 min, targeting the most modifiable risk factor for spasm and boosting procedural safety. Second, ultrasound is indispensable for preoperative assessment. For instance, one patient in the control group experienced forearm pain and subsequent swelling due to a complication with a previously undetected radial artery loop; interestingly, this patient also had a symmetrical arterial loop in the contralateral arm. Without routine preoperative ultrasound screening, this complication would have been unavoidable. Consistent with our findings, Seto et al. (24) showed ultrasound guidance reduced TRA puncture attempts (1.65 ± 1.2 vs. 3.05 ± 3.4), improved the first-pass success rate (64.8% vs. 43.9%), and reduced access time (88 ± 78 s vs. 108 ± 112 s) in 698 patients, with no significant differences observed in the rate of complications (25). But Mori et al. (26) reported that although ultrasound guidance significantly increased the procedural success rate, the puncture time and complication rate were similar.

Although there are many studies suggesting that TRA has fewer access complications than TFA (3/607, 0.5% vs. 22/635, 3.5%, *p* < 0.01) (7) and the mean time to perform the procedure through TRA was significantly shorter than the TFA (18.8 vs. 39.5 min) (27), we should pay attention to the fact that some studies found that TRA has a higher number of postoperative MRI DWI-restrictive foci than TFA in cerebral angiography, although the number of clinically symptomatic events is minimal. Carraro et al. (28) reported that of 200 consecutive diagnostic cerebral angiograms performed, 51% were performed by TRA and 49% by TFA. A total of 17.5% of TRA cerebral angiograms demonstrated at least one hyperenhanced focus on MRI DWI. In the TFA procedure, 5.2% of patients were considered positive. One patient (0.5%) in the TRA group had a minor neurologic deficit postoperatively and did not fully recover at 90 days postoperatively, whereas no neurologic deficit was observed in the TFA group. There were 0.3% (2/607) patients who experienced transient neurologic symptoms post-procedure in the TRA group in Tso et al. study (7). Our study proposes routine preoperative ultrasound as an important risk-stratification tool for this dilemma. In this study, we found that 23 of 124 patients (18.5%) had subclavian artery plaque and 9 of 124 patients (7.3%) had hypoechoic or moderately echogenic plaques that are potential embolic sources. If unstable plaques are found preoperatively by ultrasound, the operator should emphasize the risk of postoperative embolic events with the patient and family during the preoperative conversation. It is important to operate as gently as possible during the procedure to minimize catheter insertion without guidewire guidance and to reduce elective intubation of nonessential sites. It may reduce TRA-related embolic potential and improve safety compared to TFA.

Difficulties such as severe tortuosity of the right subclavian artery during catheter insertion are encountered in 6–10% of patients, increasing the risk of complications (27). The need for a specific transradial artery catheter design to prevent shape mismatch with the aortic arch and catheter kinking is obvious (3). The results of the present study on the proportions of individual aortic arch types are in agreement with the findings of Yan et al. (29). It should be noted that the mean angle of inflection of the subclavian artery was 100.2 ± 25.3° when the pigtail catheter was placed in the subclavian artery. We are developing a specialized TRA catheter to mitigate these problems, focusing on reducing the probability of kinking in the subclavian region to improve safety and efficiency. Brunet et al. (13) reported a Radial artery spasm (RAS) incidence as high as 30%. We utilized a pigtail for Simmons catheter formation and a long guidewire exchange technique to minimize RAS and artery occlusion (10, 29). Adopting a 0.035″ J-shaped guidewire facilitates operation, reduces fluoroscopy time, and avoids branch vessel entry. Therefore, ultrasound assessment and guidance proves indispensable not only for enhancing efficiency but also for improving safety.

## 5 Limitation

Our research encountered several limitations. The primary limitation of this study was its design as a non-randomized controlled trial, with patient grouping based on selection, potentially introducing selection bias. Efforts to mitigate this bias included consistent preoperative discussions with patients and families and an increased sample size. However, data collection from only one center might limit the findings’ generalizability. Additionally, monitoring complications exclusively during the 0–7 days postoperative hospitalization period may not fully capture long-term complication rates.

## 6 Conclusion

This prospective non-randomized controlled trial demonstrated that ultrasound guidance can significantly shorten surgical time and decrease difficult access rates. Subsequent study endeavors are imperative to corroborate and expand the present findings.

## Data Availability

The datasets presented in this study can be found in online repositories. The names of the repository/repositories and accession number(s) can be found in the article/supplementary material.
